# Loss of Working Life Years Due to Mortality, Sickness Absence, or Ill-health Retirement: A Comprehensive Approach to Estimating Disease Burden in the Workplace

**DOI:** 10.2188/jea.JE20190332

**Published:** 2021-07-05

**Authors:** Yosuke Inoue, Shuhei Nomura, Chihiro Nishiura, Ai Hori, Kenya Yamamoto, Tohru Nakagawa, Toru Honda, Shuichiro Yamamoto, Masafumi Eguchi, Takeshi Kochi, Toshiaki Miyamoto, Hiroko Okazaki, Teppei Imai, Akiko Nishihara, Takayuki Ogasawara, Naoko Sasaki, Akihiko Uehara, Makoto Yamamoto, Makiko Shimizu, Maki Konishi, Isamu Kabe, Tetsuya Mizoue, Seitaro Dohi

**Affiliations:** 1 Department of Epidemiology and Prevention, National Center for Global Health and Medicine, Tokyo, Japan; 2 Department of Health Policy and Management, School of Medicine, Keio University, Tokyo, Japan; 3 Department of Global Health Policy, Graduate School of Medicine, The University of Tokyo, Tokyo, Japan; 4 Institute of Global Health Policy Research (iGHP), National Center for Global Health and Medicine, Tokyo, Japan; 5 Department of Safety and Health, Tokyo Gas Co., Ltd., Tokyo, Japan; 6 Department of Global Public Health, Faculty of Medicine, University of Tsukuba, Ibaraki, Japan; 7 Division of Environment, Health and Safety, The University of Tokyo, Tokyo, Japan; 8 Hitach, Ltd., Ibaraki, Japan; 9 Furukawa Electric Co., Ltd., Tokyo, Japan; 10 NIPPON STEEL CORPORATION, Kimitsu Works, Chiba, Japan; 11 Mitsui Chemicals, Inc., Tokyo, Japan; 12 OH Support, Kanagawa, Japan; 13 Azbil Corporation, Tokyo, Japan; 14 Mitsubishi Fuso Truck and Bus Corporation, Kanagawa, Japan; 15 Hidaka Tokushukai Hospital, Hokkaido, Japan; 16 YAMAHA CORPORATION, Shizuoka, Japan; 17 East Japan Works (Keihin), JFE Steel Corporation, Kanagawa, Japan; 18 KUBOTA Corporation, Tokyo, Japan

**Keywords:** workplace, occupational health, sick leave, retirement, death

## Abstract

**Background:**

While much effort has focused on quantifying disease burden in occupational health, no study has simultaneously assessed disease burden in terms of mortality and morbidity. We aimed to propose a new comprehensive method of quantifying the disease burden in the workplace.

**Methods:**

The data were obtained from the Japan Epidemiology Collaboration on Occupational Health (J-ECOH) Study, a large-scale prospective study of approximately 80,000 workers. We defined disease burden in the workplace as the number of working years lost among the working population during a 6-year period (April 2012 to March 2018). We calculated the disease burden according to consequences of health problems (ie, mortality, sickness absence [SA], and ill-health retirement) and disease category. We also calculated the age-group- (20–39 and 40–59 years old) and sex-specific disease burden.

**Results:**

The largest contributors to disease burden in the workplace were mental and behavioural disorders (47.0 person-years lost per 10,000 person-years of working years; ie, per myriad [proportion]), followed by neoplasms (10.8 per myriad) and diseases of the circulatory system (7.1 per myriad). While mental and behavioural disorders made a greater contribution to SA and ill-health retirement compared to mortality, the latter two disorders were the largest contributors to the disease burden in the workplace due to mortality. The number of working years lost was greater among younger versus older female participants, whereas the opposite trend was observed in males.

**Conclusions:**

Our approach is in contrast to those in previous studies that focused exclusively on mortality or morbidity.

## INTRODUCTION

Employee health and well-being is one of the most important management issues, influencing employers and wider society. Work-related health problems have been linked to a decrease of 4–6% in the gross domestic product of most countries.^1^ Mortality and morbidity among employees incur both direct (eg, medical expenditures) and indirect (eg, wages lost due to sickness and social insurance claims) costs. Appropriately addressing and prioritising health issues that contribute to the disease burden in the workplace would enhance the sustainability of business and society.

Much effort has focused on quantifying the disease burden in occupational health using several indicators, such as years of potential life lost,^2^^–^^4^ disability-adjusted working life years,^5^ and economic costs.^6^^–^^12^ For example, Hanly et al^6^ estimated the disease burden associated with premature cancer deaths among adult employees by calculating the cost of lost productivity across 30 European countries.

Despite this research progress, very few attempts have been made to simultaneously assess disease burden in terms of mortality and morbidity.^8^^,^^13^ Comprehensive health indicators, including information on mortality and disability, are essential for assessing the overall health of the population.^14^^,^^15^ In occupational health, such indicators can facilitate corporate management, such as by protection of employee health and welfare, by managers, employee representatives, and occupational physicians.

We propose a new comprehensive method to quantify the disease burden in the workplace that captures both mortality and sickness absence (SA). We also report the results of application of this method to a Japanese working-age population using data from the Japan Epidemiology Collaboration on Occupational Health (J-ECOH) Study, a large-scale multicentre prospective study involving approximately 80,000 workers.

## METHODS

### Definition of disease burden in the workplace

**Figure 1.**
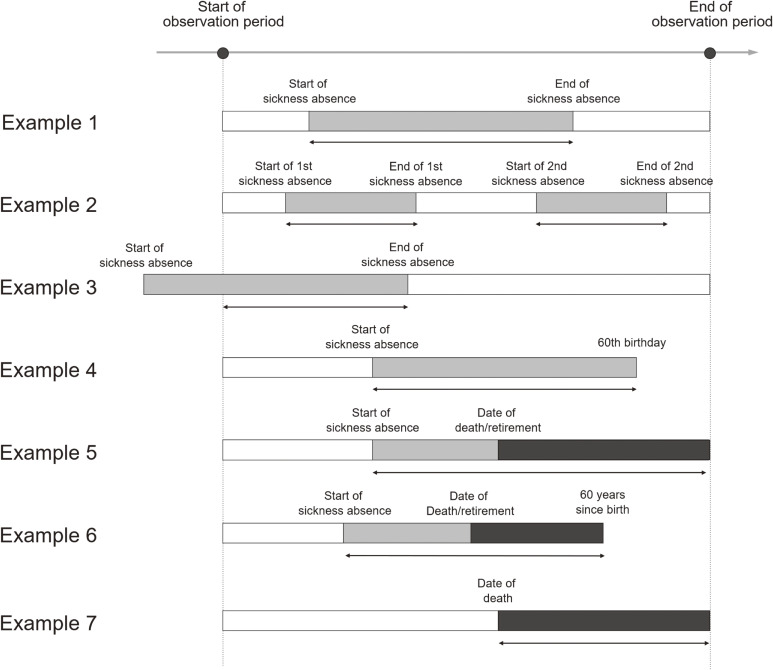
Illustration of the definition of the number of working life years lost to mortality, sickness absence, and ill-health retirement. Gray bars indicate the duration of sickness absence while black bars indicate the duration of working life years lost due to mortality or ill-health retirement. The sum of the durations indicated with arrows was defined as total working-life years lost in this study population.

We defined disease burden in the workplace as the number of working years lost among the working population during a particular observation period. Figure 1 presents several examples of how the number of working life years lost can be calculated. When an employee took SA after the start of the observation period and returned to work before its end, the disease burden due to SA of this person was calculated as the duration of SA (Example 1). If an employee took SA multiple times, the total duration of SA was considered the disease burden due to SA of this person (Example 2). If an episode of SA began before the start of the observation period, the disease burden due to SA of this person was measured from the start of the observation period to the end of SA (Example 3).

The working age was set at 20–59 years; if an employee who took SA turned 60 years old (ie, legal retirement age as of April 2012 [when we started to collect the J-ECOH data]) before the end of the observation period, the date of their 60th birthday was considered the end of SA (Example 4). Similarly, if an employee turned 20 years old during the observation period, the date of their 20th birthday was regarded as the start of the observation period (not shown).

If an employee died during the observation period, the disease burden due to mortality of this person was measured from the date of death to the end of the observation period (Examples 5 and 7). Similarly, if an employee retired (presumably due to the disease that caused SA) before the end of the observation period, the date of retirement was the end of the observation period. If an employee reached age 60 years before the end of the observation period, the end of the observation period was the 60th birthday of that person (Example 6).

### Study population

We applied our method to the Japanese working-age population of the J-ECOH. The J-ECOH is an on-going epidemiological survey of health determinants among Japanese workers across various industries (eg, electric machinery and apparatus manufacturing; steel; chemical; gas; non-ferrous metal manufacturing; automobile and instrument manufacturing; plastic product manufacturing; and health care). The study involved mainly large companies whose workers have generous employment-protection systems. For example, employees at the participating companies can use paid sick leave; they are paid over two-thirds of their salary for the maximum length of 2.5–3.9 years. Details of the cohort are available elsewhere.^16^

We used the data of employees (aged 20–59 years) at 10 companies participating in the J-ECOH, which provided employees’ information on mortality and long-term SA (LTSA; SA for ≥30 consecutive days) from April 2012 to March 2018. Information was also available on the outcome of those who took LTSA (ie, return-to-work, retirement, or death).

Information on specific diagnoses for mortality was based on death certificates, SA documents (for some of those who died during LTSA), family confirmation, and other sources. Information regarding LTSA was obtained from medical certificates endorsed by primary care physicians. Information regarding mortality and LTSA was reported by occupational physicians at each worksite. We coded the diagnoses based on the International Classification of Diseases, 10th Revision (ICD-10).

Information on the number of workers by sex and 5-year age group was available for each year from 2012 to 2017. The reported number of male and female workers aged 20–59 years in the J-ECOH was 80,769 in 2012 and 73,501 in 2017. This information was used to calculate the total number of working years in terms of person-years during the study period (ie, 409,678 person-years for males and 97,205 person-years for females).

### Calculating the number of working years lost

We divided the number of working years lost in terms of person-years by the total number of working years during the study period (also in terms of person-years) to calculate the disease burden in the workplace. Results were expressed as fractions of 10,000 (ie, per myriad [proportion]). A loss of 100 per myriad (ie, 100 person-years lost per 10,000 person-years working years) is equivalent to, for example, having 100 employees unable to provide labour service in a company of 10,000 employees during a 1-year observation period.

We calculated the fractions according to (1) consequences of health problems (ie, mortality, SA, and ill-health retirement) and (2) disease categories based on the ICD-10 chapter titles, except for Injury, poisoning and certain other consequences of external causes (S00-T98) and External causes of morbidity and mortality (V01-Y98). Information on these two chapters was first aggregated and subsequently categorised into external causes/injuries except Intentional self-harm (S00-T98/V01-Y98) and Intentional self-harm (X60-X84). We also calculated the number of working years lost by age group- and sex-specific subpopulations (ie, 20–39 year-old males; 40–59 year-old males; 20–39 year-old females; and 40–59 year-old females).

A chi-squared test was used to examine differences in the proportion of working years lost by sex and age group. Statistical analysis was conducted using Stata ver. 15.1 (College Station, TX, USA).

## RESULTS

Table 1 shows the number of working years lost to mortality, SA, and ill health retirement. The number of working years lost due to all causes during the study period was 85.9 per myriad (95% confidence interval [CI], 83.4–88.5), which comprised 40.8 per myriad (95% CI, 39.0–42.6) due to SA, 17.0 per myriad (95% CI, 15.9–18.1) due to mortality, and 28.2 per myriad (95% CI, 26.7–29.6) due to ill-health retirement.

**Table 1.** Proportion of working life years lost due to mortality, ill-health retirement, and long-term sickness absence per 10,000 population among participants in the Japan Epidemiology Collaboration on Occupational Health Study, shown by disease category^a^

Blocks	Disease categories	Total	Types of consequences		

			Sickness absence	Mortality	Ill health retirement
F00-F99	Mental and behavioural disorders	47.0 (45.1, 48.9)	26.4 (25.0, 27.9)	0.1 (0.0, 0.2)	20.5 (19.3, 21.8)
C00-D48	Neoplasms	10.8 (9.9, 11.8)	3.2 (2.8, 3.8)	6.5 (5.9, 7.3)	1.0 (0.8, 1.4)
I00-I99	Diseases of the circulatory system	7.1 (6.4, 7.9)	2.2 (1.9, 2.7)	4.0 (3.5, 4.6)	0.9 (0.6, 1.2)
S00-T98	External causes/injuries except intentional self-harm (X60-X84)	3.9 (3.3, 4.4)	1.6 (1.3, 2.0)	1.2 (1.0, 1.6)	1.0 (0.8, 1.3)
V01-Y98					
M00-M99	Diseases of the musculoskeletal system and connective tissue	3.6 (3.1, 4.1)	2.1 (1.7, 2.6)	0.1 (0.0, 0.2)	1.4 (1.1, 1.7)
X60-X84	Intentional self-harm	3.3 (2.8, 3.9)	0.0 (0.0, 0.1)	3.3 (2.8, 3.9)	0.0 (0.0, 0.1)
G00-G99	Diseases of the nervous system	3.3 (2.8, 3.8)	2.0 (1.6, 2.4)	0.0 (0.0, 0.1)	1.3 (1.0, 1.6)
K00-K93	Diseases of the digestive system	1.5 (1.2, 1.9)	0.5 (0.4, 0.8)	0.7 (0.4, 0.9)	0.4 (0.2, 0.6)
R00-R99	Symptoms, signs and abnormal clinical and laboratory findings, not elsewhere classified	1.2 (0.9, 1.6)	0.5 (0.3, 0.7)	0.4 (0.3, 0.7)	0.3 (0.2, 0.5)
O00-O99	Pregnancy, childbirth and the puerperium	1.0 (0.7, 1.3)	0.6 (0.4, 0.9)	0.0 (0.0, 0.1)	0.4 (0.2, 0.6)
J00-J99	Diseases of the respiratory system	0.8 (0.6, 1.1)	0.3 (0.2, 0.5)	0.3 (0.2, 0.5)	0.2 (0.1, 0.3)
E00-E99	Endocrine, nutritional and metabolic diseases	0.6 (0.4, 0.8)	0.3 (0.2, 0.5)	0.0 (0.0, 0.1)	0.3 (0.2, 0.5)
N00-N99	Diseases of the genitourinary system	0.5 (0.3, 0.7)	0.2 (0.1, 0.4)	0.2 (0.1, 0.4)	0.1 (0.0, 0.2)
H00-H59	Diseases of the eye and adnexa	0.4 (0.2, 0.6)	0.2 (0.1, 0.4)	0.0 (0.0, 0.1)	0.1 (0.1, 0.3)
H60-H95	Diseases of the ear and mastoid process	0.3 (0.2, 0.5)	0.2 (0.1, 0.3)	0.0 (0.0, 0.1)	0.1 (0.0, 0.2)
A00-B99	Certain infectious and parasitic diseases	0.3 (0.1, 0.4)	0.2 (0.1, 0.3)	0.1 (0.0, 0.2)	0.0 (0.0, 0.1)
L00-L99	Diseases of the skin and subcutaneous tissue	0.2 (0.1, 0.3)	0.1 (0.0, 0.2)	0.0 (0.0, 0.1)	0.1 (0.0, 0.2)
Q00-Q99	Congenital malformations, deformations and chromosomal abnormalities	0.1 (0.1, 0.3)	0.0 (0.0, 0.1)	0.0 (0.0, 0.1)	0.1 (0.0, 0.3)
D50-D89	Diseases of the blood and blood-forming organs and certain disorders involving the immune mechanism^b^	0.0 (0.0, 0.1)	0.0 (0.0, 0.1)	0.0 (0.0, 0.1)	0.0 (0.0, 0.1)
Z00-Z99	Factors influencing health status and contact with health services^b^	0.0 (0.0, 0.1)	0.0 (0.0, 0.1)	0.0 (0.0, 0.1)	0.0 (0.0, 0.1)

	Total	85.9 (83.4, 88.5)	40.8 (39.0, 42.6)	17.0 (15.9, 18.1)	28.2 (26.7, 29.6)

^a^Disease burden was calculated by dividing the number of working years lost in terms of person-years by the total number of working years during the study period (also in terms of person-years) and results are expressed as fractions of 10,000 (ie, per myriad [proportion]).

^b^Numbers of working life years lost to factors influencing health status and contact with health services (Z00-Z99) and diseases of the blood and blood-forming organs and certain disorders involving the immune mechanism (D50-D89) were 0.039 and 0.039, respectively.

Mental and behavioural disorders (F00-F99) made the largest contribution to the total number of working years lost—47.0 per myriad (95% CI, 45.1–48.9), which was equivalent to 54.7% of the total disease burden, were lost to this disease category. More specifically, SA, mortality, and ill-health retirement contributed 26.4 (95% CI, 25.0–27.9), 0.1 (95% CI, 0.0–0.2), and 20.5 (95% CI, 19.3–21.8) per myriad lost, respectively. The other significant contributors were neoplasms (C00-D48; 10.8, 95% CI, 9.9–11.8) and diseases of the circulatory system (I00-I99; 7.1, 95% CI, 6.4–7.9). The disease burden due to these disease groups was characterised as a higher contribution to disease burden associated with mortality vs disease burden associated with SA or ill-health retirement (6.5 per myriad [95% CI, 5.9–7.3] for neoplasms and 4.0 per myriad [95% CI, 3.5–4.6] for diseases of the circulatory system).

In analyses by sex and age group, the number of working years lost was greater among younger (aged 20–39 years) than older (aged 40–59 years) female participants (108.6 per myriad [95% CI, 98.8–119.0] vs 75.5 per myriad [95% CI, 68.5–83.0], *P* < 0.001), whereas the opposite trend was observed in male participants (70.0 per myriad [95% CI, 66.0–74.1] vs 95.0 per myriad [95% CI, 91.2–98.9], *P* < 0.001). The number of working years lost due to mental and behavioural disorders was higher among younger than older participants, and the difference was more pronounced in females (82.8 per myriad [95% CI, 74.3–91.9] vs 27.6 per myriad [95% CI, 23.4–32.3] in females, *P* < 0.001; and 52.2 per myriad [95% CI, 48.8–55.8] vs 41.9 per myriad [95% CI, 39.3–44.5] in males, *P* < 0.001). Neoplasms and diseases of the circulatory system were responsible for greater numbers of working life years lost among older vs younger participants of both sexes. External causes/injuries did not exhibit such distinct age-related differences. Among 20–39 year-old female participants, the second largest contributor to the number of working years lost was pregnancy-related disorders (O00-O99).

## DISCUSSION

We propose a simple method of quantifying disease burden in the workplace, in which disease burden is calculated as the number of working years lost to mortality, SA, and ill-health retirement among employees. The largest contributor to disease burden in the workplace was mental and behavioural disorders (F00-F99; 47.0 person-years lost per 10,000 person-years of working years), followed by neoplasms (C00-D48; 10.8), diseases of the circulatory system (I00-I99; 7.1), and external causes/injuries excluding intentional self-harm (S00-T98/V01-Y98; 3.9), and diseases of the musculoskeletal system and connective tissue (M00-M99; 3.6).

Mental and behavioural disorders (F00-F99) made the largest contribution to the total number of working years lost; the contribution to SA and ill-health retirement was greater than that to mortality. The impact was even larger when we accounted for the disease burden associated with intentional self-harm (ie, suicide). While several previous studies identified other diseases as the largest contributors to the disease burden associated with occupational morbidity; eg, circulatory diseases^17^ and injuries/external causes,^11^ this may be due to differences in the outcomes used to quantify the disease burden, the definition of SA (eg, the number of SA days included in the analysis), or the sociodemographic characteristics of the participants (eg, age, sex, and socioeconomic status).

The greater number of working years lost due to mental and behavioural disorders among the younger versus older age groups was not in line with the 2017 Patient Survey of the Japan Ministry of Health, Labour, and Welfare.^18^ For example, treatment rates for mental and behavioural disorders among 20–24 and 50–59 year-old males were 159 and 592 per 10,000 population. The corresponding figures for female participants were 192 and 491, respectively. This inconsistency may be because our finding resulted from the survival effect (ie, those with mental disorders are more likely to leave the company at a younger age). It is also possible that in our dataset, younger workers suffered from lack of job control more than older employees (which may be linked to a higher disease burden for mental disorders); however, previous studies have reported conflicting findings on the association between age and job control/decision latitude.^19^^,^^20^ The higher disease burden among younger females versus younger males is in line with prior reports,^16^^,^^18^^,^^21^ and the opposite trend among the older age group is likely due to a disproportionate survival effect among female versus male employees.

Our finding that neoplasms were the largest contributor to the disease burden in the workplace due to mortality (ie, 6.5 of 17.0 per myriad for total working years lost to mortality) is in accordance with the work of Yoshida et al,^2^ who reported that malignant neoplasms are the leading cause of potential life years lost due to mortality in a working population in Japan. Leigh,^8^ who estimated the United States’ national costs of occupational injury and illness, reported that cancer accounted for 38.1% of the total deaths and 23.2% of the medical costs associated with mortality (second highest, after circulatory diseases). We extended these previous studies by accounting for not only the disease burden associated with mortality but also disease burden associated with SA and ill-health retirement. This may inform future efforts to mitigate the disease burden associated with cancer, such as by anti-smoking campaigns and cancer screening.

The age-related increase in the number of working life years lost due to neoplasms and circulatory disorders was in line with the age-specific incidence in a prior report.^22^^,^^23^ Compared to mental and behavioural disorders, neoplasms and circulatory disorders made a greater contribution to the number of working life years lost to mortality. This may explain the above-mentioned age-related increase in the number of working life years lost due to neoplasms and circulatory disorders.

We did not find an age-related increase in the disease burden associated with self-harm, which contradicts previous reports,^24^^,^^25^ possibly due to the survival effect.^24^ However, we detected a notable sex difference. Specifically, the disease burden associated with death by suicide was larger among males versus females. This is in accordance with Amagasa et al,^26^ who investigated the characteristics of 22 cases of work-related suicide (ie, “karojisatsu”) in Japan and reported only one female case. It is also in line with previous reports that the number of suicides was higher among males versus females in the general population of Japan.^24^^,^^25^ Given that Ahn et al^24^ did not report a pronounced sex-related difference in suicide methods (ie, violent vs non-violent) in Japan, future studies should focus on the mechanisms underlying this sex-related difference (eg, in the proportion of those engaged in overtime work^27^).

While the Global Burden of Disease (GBD) study reported that musculoskeletal disorders accounted for 18.0–29.7% of disability adjusted life years among 20–59 years old in Japan,^28^ our study showed that musculoskeletal disorders contributed to approximately 5% of disease burden associated with morbidity (ie, SA and ill-health retirement). While this inconsistency seemed to have arisen from the difference in source of information used to define morbidity (the GBD study used information collected via population-representative health surveys or hospital-based registries), it is also possible that our findings might have resulted from our definition of SA (ie, consecutive 30 days of medically-certified SA). The disease burden due to musculoskeletal disorders in our study might occupy a larger fraction of disease burden if we use information on short-term or self-reported SA. As a result of using long-term SA to define morbidity, our study consequently assessed disease burden caused by serious health conditions such that they have substantial damage to business sustainability.

It is of note that the second leading contributor to the number of working years lost among the younger-females group was pregnancy-related disorders (Table 2). Caution should be exercised when interpreting the results in Table 1, as the J-ECOH study included a smaller percentage of female workers than in the general population and the disease burden associated with pregnancy-related disorders was seemingly minor. Such disorders may be higher in companies with a higher percentage of younger female workers. Managers should be aware of this disease burden in the workplace and female workers with such disorders should be supported and protected (eg, making practicable adjustments to their roles) to enable them to continue work or be reinstated following illness.

**Table 2.** Proportion of working life years lost per 10,000 population among participants in the Japan Epidemiology Collaboration on Occupational Health Study, shown by sex and age group (20–39 and 40–59 years old)^a,b^

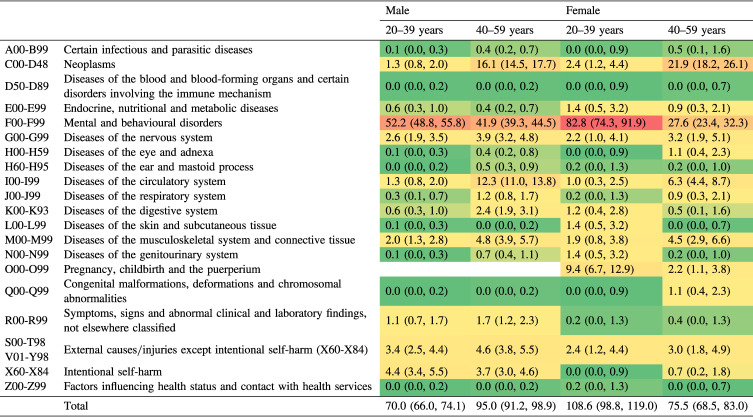

^a^Disease burden was calculated by dividing the number of working years lost in terms of person-years by the total number of working years during the study period (also in terms of person-years) and results are expressed as fractions of 10,000 (ie, per myriad [proportion]). The observation periods were 42,234 person-years for 20–39 year old males, 226,094 person-years for 40–59 year old males, 28,062 person-years for 20–39 year old females, and 43,599 person-years for 40–59 year old females.

^b^Values are colored using a continuous color gradient; the maximum, median and minimum values were colored red (the RGB color 248 105 107), yellow (255 235 132) and green (99 190 123).

This study had several strengths. First, the approach contrasts those in previous studies that focused exclusively on mortality^2^^,^^6^ or morbidity^3^^–^^5^^,^^9^^–^^12^^,^^17^ and enhances our understanding of the disease burden in the workplace. Second, the method uses routinely available data, such as long-term SA and mortality. In contrast to self-reported information, which was used in several prior works, the information used in our study is objective and not subject to recall bias. Third, the approach is easy to use in occupational health, and will help managers and occupational physicians understand the disease burden in their company; they can understand the magnitude of disease burden relative to other companies as well as temporal changes within their companies.

Several issues should be borne in mind when interpreting the results. First, this study used information on long-term SA (ie, ≥30 consecutive days) to quantify the disease burden associated with morbidity. The results would have been different if we had possessed information on short-term SA. In addition, we did not account for the extent to which each workplace was burdened by presenteeism.^29^^,^^30^ Second, those who retired might have started to work for another company during the study period; thus, the disease burden related to retirement may have been overestimated. Third, we confined our study participants to those aged less than 60 years old (ie, the general retirement age in Japan), and we did not account for disease burden among older age groups. Fourth, the J-ECOH study involved mainly large companies whose workers have generous employment-protection systems and so caution should be exercised when interpreting our findings. In addition, we did not collect information from workers in some industries with an elevated level of occupational hazard (eg, construction).^31^ Fifth, there are several variables that could have been incorporated to make between-company comparison more relevant (eg, socio-economic status and sex ratio of participants). For example, the disease burden of pregnancy-related disorders was likely influenced by the sex ratio of the participants.

### Conclusions

We propose a simple method of quantifying the disease burden in the workplace. Using information collected by the J-ECOH study, we showed that mental and behavioural disorders made the greatest contribution to the total number of working years lost, followed by neoplasms and diseases of the circulatory system.

Employers might be more concerned with the disease burden associated with SA compared to that associated with death or retirement, which do not require continued support once employees leave the company. However, the numbers of working years lost calculated in this study represents the extent to which the employers lose a healthy labour force, which could have been prevented by better occupational health and safety management systems. This is a great loss not only for the employer but also for wider society. Each company can use this tool to quantify disease burden, which should serve as a scientific base for the expansion of health promoting activities.
